# Down-regulation of cholinergic signaling in the habenula induces anhedonia-like behavior

**DOI:** 10.1038/s41598-017-01088-6

**Published:** 2017-04-18

**Authors:** Seungrie Han, Soo Hyun Yang, Jin Yong Kim, Seojung Mo, Esther Yang, Ki Myung Song, Byung-Joo Ham, Naguib Mechawar, Gustavo Turecki, Hyun Woo Lee, Hyun Kim

**Affiliations:** 1 Department of Anatomy, College of Medicine, Korea University, Seoul, 136-705 Korea; 2 Department of Psychiatry, College of Medicine, Korea University, Seoul, 136-705 Korea; 3 Department of Psychiatry, McGill University, Douglas Mental Health University Institute, Montreal, QC H4H 1R3 Canada

## Abstract

Dysfunction of cholinergic signaling in the brain has long been believed to be associated with depressive disorders. However, the functional impact of habenular cholinergic signaling on the specified depressive behaviors is not well understood. Here, we demonstrated that the expression levels of cholinergic signaling genes (CHAT, VACHT, CHT, CHRNA3, CHRNB3 and CHRNB4) were down-regulated in a chronic restraint stress (CRS) rat model of depression, in which rats display depression-like behaviors such as anhedonia and mood despair. Moreover, knockdown of CHAT in the rat habenula was sufficient to evoke anhedonia-like behavior. The anhedonia-like behavior induced by CHAT knockdown was not reversed by chronic administration of the selective serotonin reuptake inhibitor fluoxetine. To determine whether habenular cholinergic signaling is associated with regulation of dopamine neurons in the ventral tegmental area (VTA) and serotonin neurons in the dorsal raphe nucleus (DRN), we used CHAT::cre transgenic mice expressing the Designer Receptors Exclusively Activated by Designer Drugs (DREADD). Pharmacogenetic activation of habenular cholinergic neurons induces the excitation of dopamine neurons in the VTA and reduces the immunoreactivity of 5-hydroxytryptamine (5-HT) in the DRN. Habenular cholinergic gene down-regulation was recapitulated in the postmortem habenula of suicide victims diagnosed with major depressive disorder (MDD).

## Introduction

Major depressive disorder (MDD) is a serious psychiatric disorder that is frequently associated with suicide attempts and a major contributor to the global burden of disease^1^. Cholinergic transmission in the brain has long been thought to underlie depression endophenotypes^2–4^. Enhancement of extracellular acetylcholine (ACh) level through administration of acetylcholinesterase inhibitors can elicit depression symptoms in both humans and rodents^5,6^. Moreover, inhibition of nicotinic acetylcholine receptors (nAChRs) or muscarinic acetylcholine receptors (mAChRs) ameliorates depression symptoms^7,8^. Although these lines of evidence clearly suggest that ACh signaling contributes to depression, it is not yet clear which specific cholinergic neuronal populations are responsible for specific depression symptoms.

The habenula of the epithalamus anatomically and functionally links the forebrain with the midbrain structures that are involved in the release of dopamine (i.e., the substantia nigra pars compacta and VTA) and serotonin (i.e., raphe nucleus)^9,10^. The habenula can be subdivided into the medial habenula (MHb) and the lateral habenula (LHb). Several lines of evidence from studies in animal models and humans suggest that the MHb plays a major role in nicotine addiction^11–13^, whereas dysregulation of the LHb is likely to be involved in several psychiatric disorders, including depression. Deep brain stimulation of the LHb produces marked remission of therapy-refractory depression in patients^14^ and congenitally learned helplessness in rats^15^. However, the role of the MHb in the pathophysiology of depression remains unclear.

Considering that the habenular cholinergic projection through the fasciculus retroflexus (fr) is one of the major cholinergic pathways in the brain^16^, and that dysfunction of central cholinergic signaling is critical for depression^5–8^, we asked whether the activity of habenular cholinergic signaling is associated with depression.

In the present study, we demonstrate that cholinergic genes in the habenula are down-regulated in both rat models of depression and human MDD patients. Selective reduction of habenular cholinergic signaling induce the anhedonia-like behavior, which may be associated with dopamine and serotonin neuronal activities in the VTA and DRN, respectively. Notably, reduced MHb cholinergic signaling-induced anhedonia-like symptom is not reversed by chronic administration of fluoxetine. Our analyses indicate that abnormal MHb cholinergic signaling is associated with the pathogenesis of depression.

## Results

### Development of Animal Model of Depression

To analyze depression-related changes of habenular cholinergic gene expression at the level of mRNA, we developed classic chronic restraint stress (CRS) model of depression. Repeated exposure to stressful events, which is a risk factor in the etiology of depression, is sufficient to drive transcriptional changes in the habenula (Supplementary Figure S1a). Compared with animals that were not subjected to the stress procedure (non-stressed, NS), all stressed animals exhibited greater body weight loss (body weight gain after 2 weeks of stress; NS, 97.667 ± 3.989 g; CRS, 50.400 ± 10.289 g; *P* = 0.001; Supplementary Figure S1b) higher plasma corticosterone levels (NS, 125.771 ± 46.434 mg/mL; CRS, 591.466 ± 99.732 mg/mL; *P* = 0.002; Supplementary Figure S1c), and greater adrenal gland weight (NS, 127.661 ± 8.783; CRS, 180.106 ± 12.075; expressed as μg/g of body weight; *P* = 0.006; Supplementary Figure S1d). We next tested whether rats subjected to CRS showed depression-like behaviors in the sucrose preference test (SPT) and forced swim test (FST). The results of the SPT revealed that the rats in the CRS group consumed a significantly lower amount of the sucrose solution than did rats in the NS group (sucrose preference expressed as a percentage of the total fluid intake: NS, 85.083 ± 2.379; CRS, 61.875 ± 5.991; *P* = 0.002; Supplementary Figure S1e), demonstrating an anhedonia-like phenotype in the CRS model of depression. The CRS rats also displayed despair-like behavior as they showed an increased immobility in the FST (immobility time: NS, 120.00 ± 12.429 s; CRS, 170.00 ± 14.500 s; *P* = 0.013; Supplementary Figure S1f). These data validated the CRS model as a tool for determining the effects of chronic stress-induced depression on gene expression.

### Habenular Cholinergic Genes are Down-regulated in the CRS Rat Model

**Figure 1:**
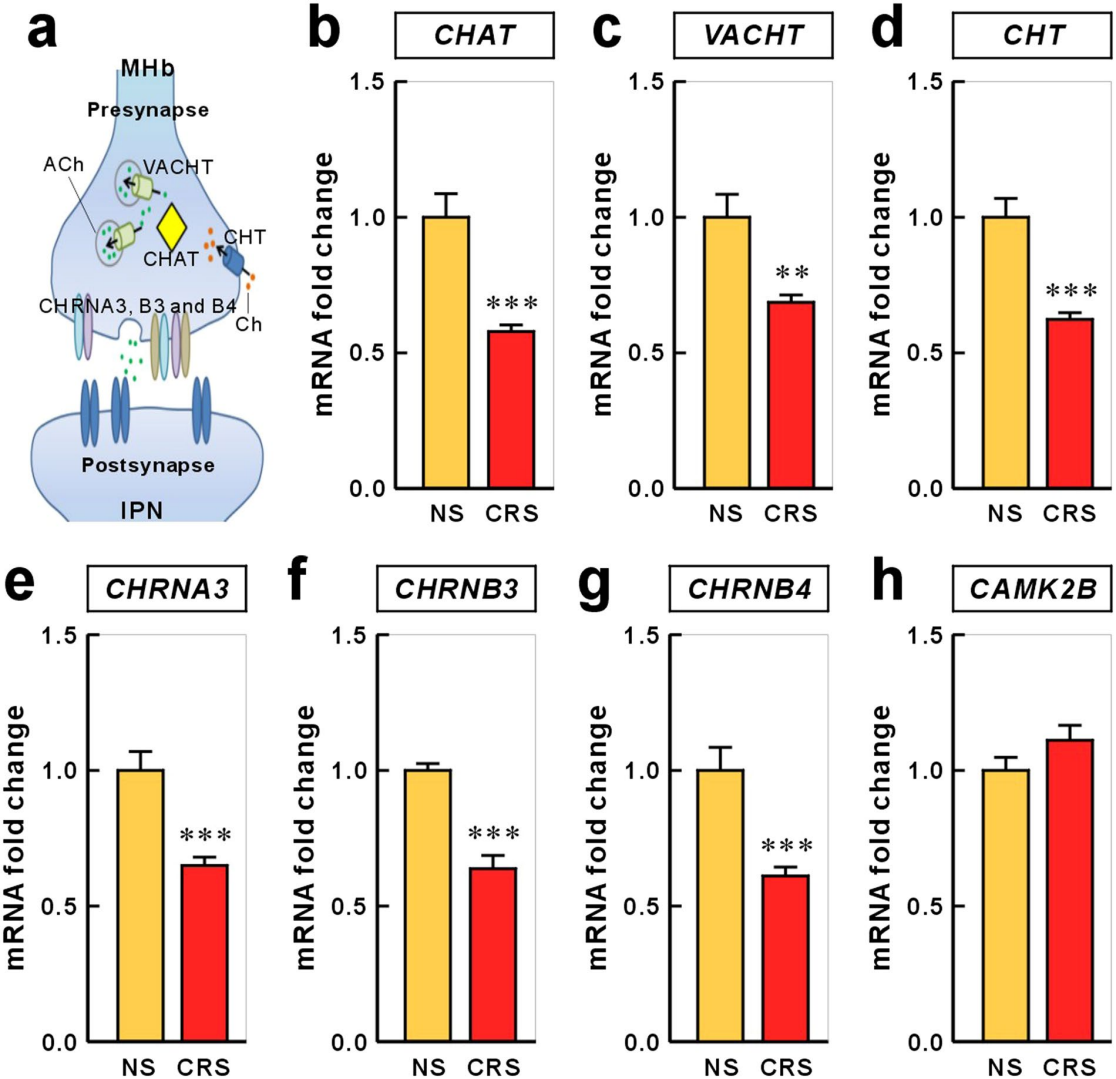
Effect of chronic restraint stress on the expression of cholinergic system genes in the rat habenula. (**a**) Schematic diagram of a generalized cholinergic synapse. The choline transporter protein (CHT) delivers choline into the cytoplasm, where choline acetyltransferase (CHAT) catalyze the transfer of an acetyl group from the coenzyme, acetyl Co-A, to choline, generating acetylcholine, and the vesicular acetylcholine transporter (VACHT) packs acetylcholine into the vesicle. Presynaptic nicotinic acetylcholine receptors (nAChR; CHRNA3, CHRNB3 and CHRNB4) modulate the release of neurotransmitter. (**b**–**h**) qRT-PCR was used to quantify mRNA expression levels of six cholinergic system genes in the habenula of rats exposed to CRS. The mRNA expression levels of CHAT, VACHT, CHT, CHRNA3, CHRNB3, and CHRNB4 were significantly reduced by CRS. Values for each individual gene were normalized to the mean of the reference gene GAPDH. CAMK2B was not changed by CRS. Data represent mean ± SEM. NS, n = 4; CRS, n = 4; *P < 0.05, **P < 0.01, ***P < 0.001, Mann–Whitney *U*-test.

We utilized this CRS animal model of depression to measure the mRNA levels of the target genes in the habenula. ACh neurotransmission occurs through Ca^2+^–stimulated docking, fusion, and fission of the vesicles at a cholinergic presynaptic nerve terminal membrane (Fig. 1a). ACh is synthesized by choline acetyltransferase (CHAT), and is loaded into synaptic vesicles by the vesicular acetylcholine transporter (VACHT). The choline that is transported back into the presynaptic neuron by the choline transporter (CHT) is available for the synthesis of additional ACh^17^. Presynaptic nAChRs are known to mediate positive feedback to enhance synaptic transmission^18,19^. In the habenulo-interpeduncular nucleus pathway, ACh release is mediated by habenular presynaptic nAChRs, consisting of CHRNA3, CHRNB3, and CHRNB4^20,21^. We found that the gene expression levels for CHAT, VACHT, CHT, CHRNA3, CHRNB3, and CHRNB4 were all significantly reduced in the habenula of CRS rats compared with those of NS rats (NS vs. CRS expressed as fold change: CHAT, 1.000 ± 0.087 vs. 0.579 ± 0.024, *P* = 0.0004; VACHT, 1.000 ± 0.085 vs. 0.686 ± 0.027, *P* = 0.0033; CHT, 1.000 ± 0.069 vs. 0.624 ± 0.025, *P* = 0.0002; CHRNA3, 1.000 ± 0.070 vs. 0.649 ± 0.031, *P* = 0.0004; CHRNB3, 1.000 ± 0.025 vs. 0.638 ± 0.049, *P* = 0.0001; CHRNB4, 1.000 ± 0.085 vs. 0.611 ± 0.033, *P* = 0.0008; Fig. 1b–g), which is consistent with our gene expression findings in another animal model of depression, learned helpless rats (Supplementary Figure S2). However, CAMK2B expression was not significantly altered (1.000 ± 0.048 vs. 1.136 ± 0.075, *P* = 0.1484; Fig. 1h). RNA *in situ* hybridization further confirmed the reduced mRNA expression of cholinergic genes in the ventral MHb of the CRS-subjected rats (Supplementary Figure S3). To validate the qPCR results we further examined the protein expression for a subset of selected genes, CHAT and VACHT, with immunohistochemistry and western blot. The immunoreactivities of CHAT and VACHT were decreased more pronouncedly in CRS group than in NS group, consistent with corresponding gene expression profiling as verified by qPCR analysis (Supplementary Figure S4).

### Knockdown of Habenular CHAT Drives Anhedonia-like Behavior

**Figure 2:**
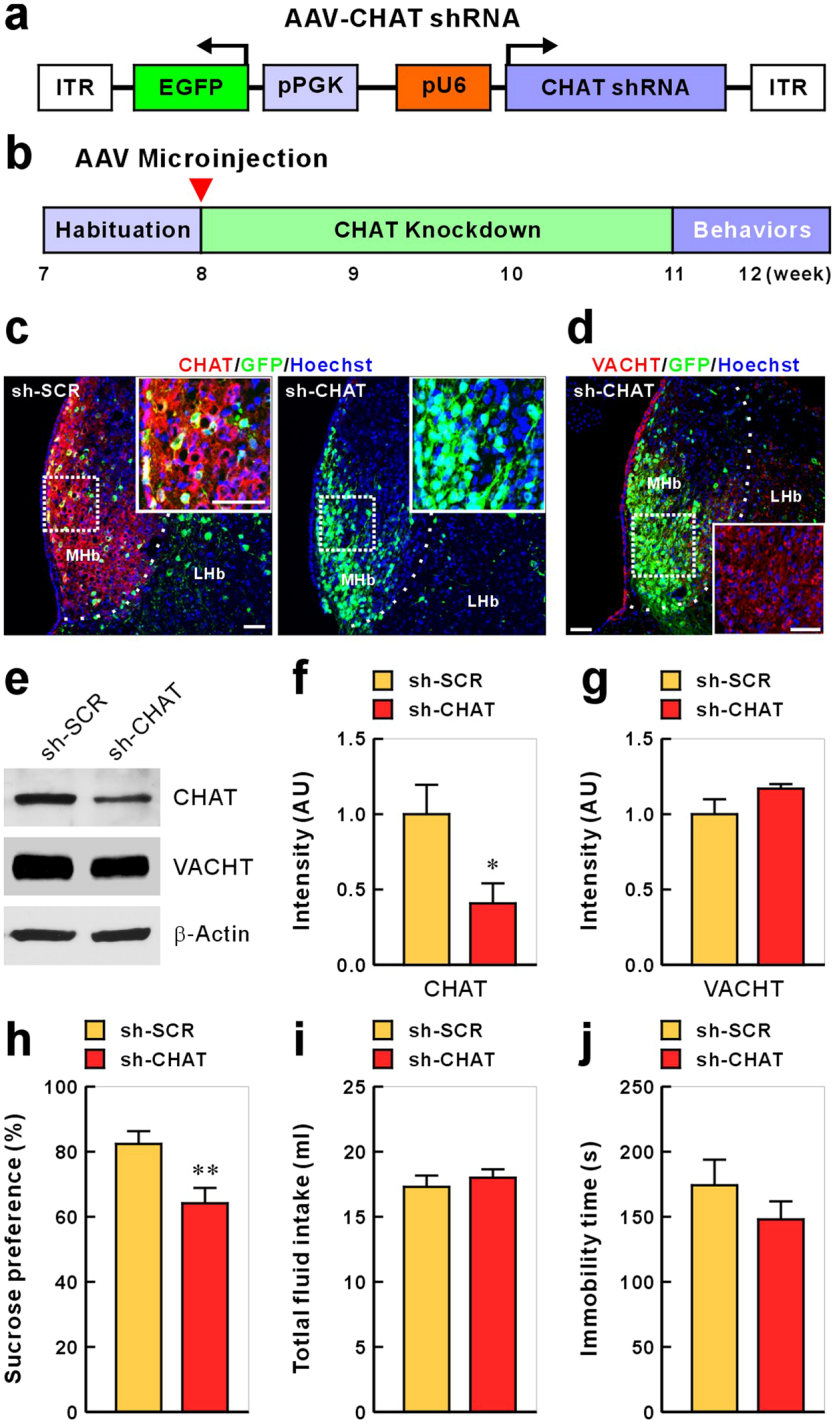
Effects of CHAT knockdown in medial habenula on depression-like behaviors. (**a**) Schematic representation of the AAV vector engineered to induce CHAT knockdown. (**b**) Experimental paradigm for behavioral testing of rats infected with the virus. (**c**) Representative photomicrographs of Hb slices from the rats injected with AAV-sh-SCR (control) or AAV-sh-CHAT (CHAT knockdown). CHAT protein expression was visualized as red immunofluorescence. Cell nuclei and viral infection were visualized using Hoechst staining (blue) and GFP (green), respectively. The inset box shows a higher magnification of an infected MHb region. None of the neurons infected with the AAV-sh-CHAT showed CHAT expression. Scale bar, 50 μm. (**d**) VACHT expression is not affected by AAV-sh-CHAT. Hb neurons injected with AAV-sh-CHAT were immunostained for VACHT (red) and EGFP (green). Scale bar, 50 μm. (**e**) CHAT and VACHT western blots from the Hb of rats injected with AAV-sh-CHAT. Rats were injected with AAV-sh-SCR (control) or AAV-sh-CHAT, which were expressed for 3 weeks before western blot analysis. (**f**) Quantification of the effects of shRNA-mediated CHAT knockdown. Data represent mean ± SEM (sh-SCR, n = 4 rats; sh-CHAT, n = 4; *P < 0.05, Student’s *t*-test). (**g**) VACHT expression is not affected by AAV-sh-CHAT. Data represent mean ± SEM (sh-SCR, n = 4 rats; sh-CHAT, n = 4) (**h**–**j**) Behavioral effects of expressing AAV-sh-CHAT in the MHb on sucrose preference (**h**), total fluid intake during SPT test (**i**), and forced swim (**j**). Data represent mean ± SEM (sh-SCR, n = 20; sh-CHAT, n = 27; **P < 0.01, Student *t*-test).

We next investigated whether the selective suppression of the habenular cholinergic circuit mimicked depression-related behaviors. Choline acetylating activity in the brain is not detectable in CHAT knockout mice^22^, indicating that CHAT is the sole enzyme responsible for the biosynthesis of ACh. Thus, we used RNAi for specifically knocking down the expression of CHAT to inactivate cholinergic neurons. We first constructed a viral vector, adeno-associated virus 2/9 (AAV2/9), to express small interfering RNA (siRNA) targeted against CHAT in the MHb (Fig. 2a and b). We confirmed that, compared with siRNA having a scrambled siRNA target sequence (sh-SCR), the siRNA specifically targeting the CHAT transcript effectively reduced CHAT protein expression (sh-SCR vs. sh-CHAT, intensity expressed in arbitrary units: 1.000 ± 0.208 vs. 0.414 ± 0.142, *P* = 0.043; Fig. 2c,e and f). Although the VACHT gene is localized to the first intron of the CHAT gene^23^, VACHT expression was not affected by sh-CHAT (1.000 ± 0.106 vs. 1.175 ± 0.027, *P* = 0.386; Fig. 2d,e and g), indicating that sh-CHAT specifically down-regulated CHAT expression. We then tested the effects of a habenula-specific CHAT knockdown on depression-like behavioral phenotypes. Reduced expression of CHAT in the MHb of control rats led to decreased consumption of the sucrose solution in the SPT (sucrose preference expressed as a percentage of the total fluid intake: 82.400 ± 3.916 vs. 64.148 ± 4.726, *P* = 0.007; Fig. 2h) with no difference in total intake of liquid (water plus sucrose solution: 17.30 ± 0.869 mL vs. 18.00 ± 0.645 mL, *P* = 0.696; Fig. 2i). Importantly, no effect on immobility time in the FST was detected (174.25 ± 19.636 s vs. 147.963 ± 13.197 s, *P* = 0.255; Fig. 2j). Additionally, similar reduction of the body weight gain as CRS rats was observed in rats infected with AAV-sh-CHAT (Supplementary Figure S5). Together, these data suggest that inactivation of habenular cholinergic signaling induces anhedonia-like behavior but not despair.

### Anhedonia-like Behavioral Effect of CHAT Knockdown Was Not Reversed with Fluoxetine Treatment

**Figure 3:**
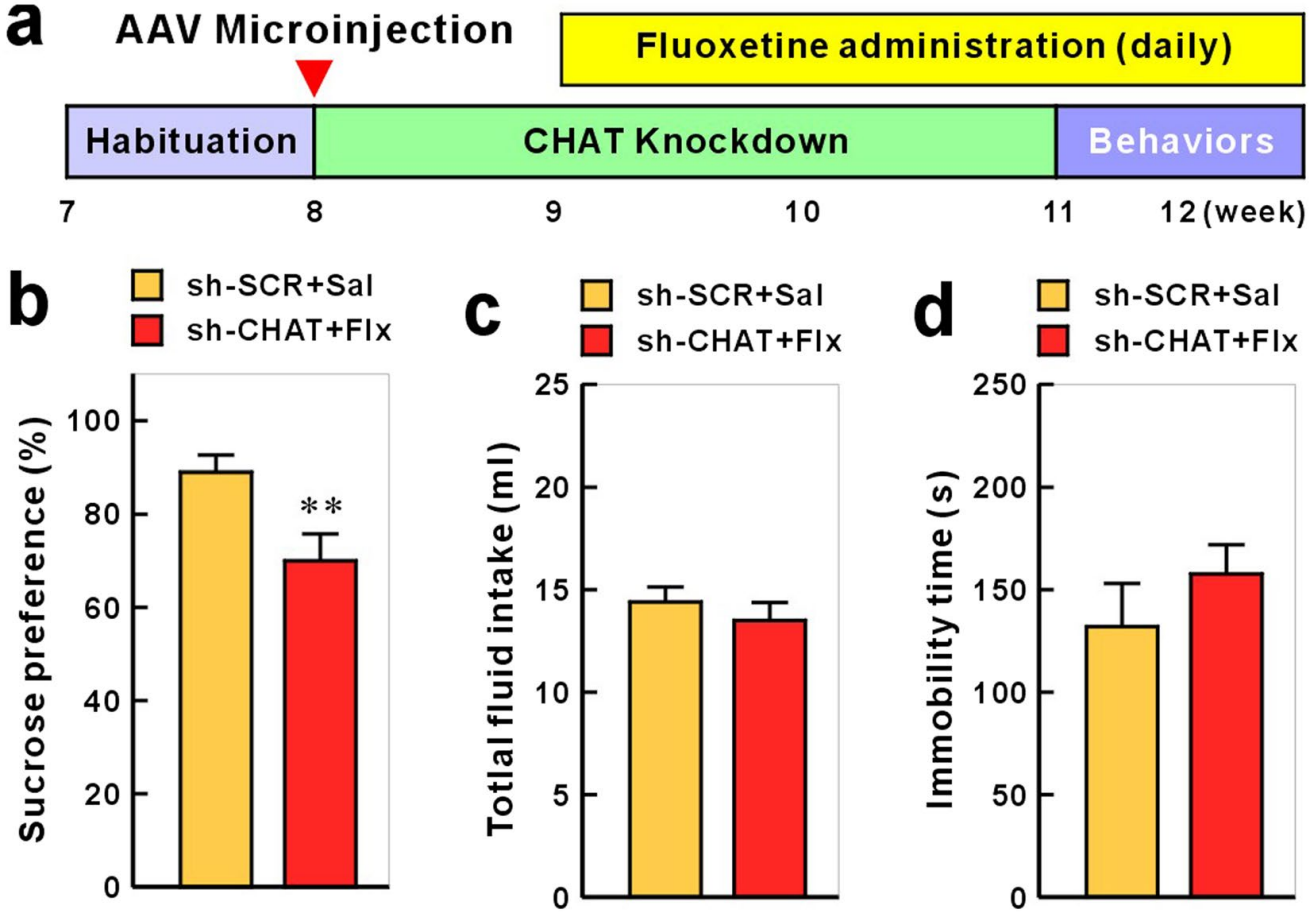
Fluoxetine does not reverse the anhedonia-like behavior induced by CHAT knockdown. (**a**) Rats were infused with AAV-sh-SCR or AAV-sh-CHAT and chronically treated with saline or fluoxetine (5 mg/kg) from one week after AAV infections throughout the experiment. Chronic administration of fluoxetine did not recover the effects of AAV-sh-CHAT infusion in the sucrose preference test (**b**), though no changes of total fluid consumption (**c**) or immobile time in the FST (**d**). Data represent mean ± SEM (sh-SCR + Sal, AAFV-sh-SCR + saline, n = 9 rats; sh-CHAT + Flx, AAV-sh-CHAT + fluoxetine, n = 15; **P < 0.01, Student’s *t*-test).

To evaluate whether the anhedonia-like behavioral effect of habenular CHAT knockdown was sensitive to antidepressant administration, rats injected with AAV-sh-CHAT into the MHb were chronically treated with fluoxetine (10 mg/kg) and subjected to the SPT or FST (Fig. 3a). Chronic administration of fluoxetine did not reverse the MHb CHAT knockdown-induced reduction in sucrose preference (Fig. 3b). There was no difference in the total fluid consumption (Fig. 3c) and immobile time in the FST (Fig. 3d). Additionally, chronic treatment with fluoxetine in the CHAT knockdown rats showed severely reduced weight gain (Supplementary Figure S6), compared with difference between control and CHAT knockdown (Supplementary Figure S5). This chronic fluoxetine administration-induced weight loss is consistent with clinical report that weight loss is associated with the use of fluoxetine^24^. These results suggest that anhedonia-like behavior induced by weakened MHb cholinergic signaling may be fluoxetine treatment-refractory symptom.

### Activation of Habenular Cholinergic Neurons Excites Dopamine Neurons in the VTA and Reduces 5-HT-Immunoreactivity in the DRN

**Figure 4:**
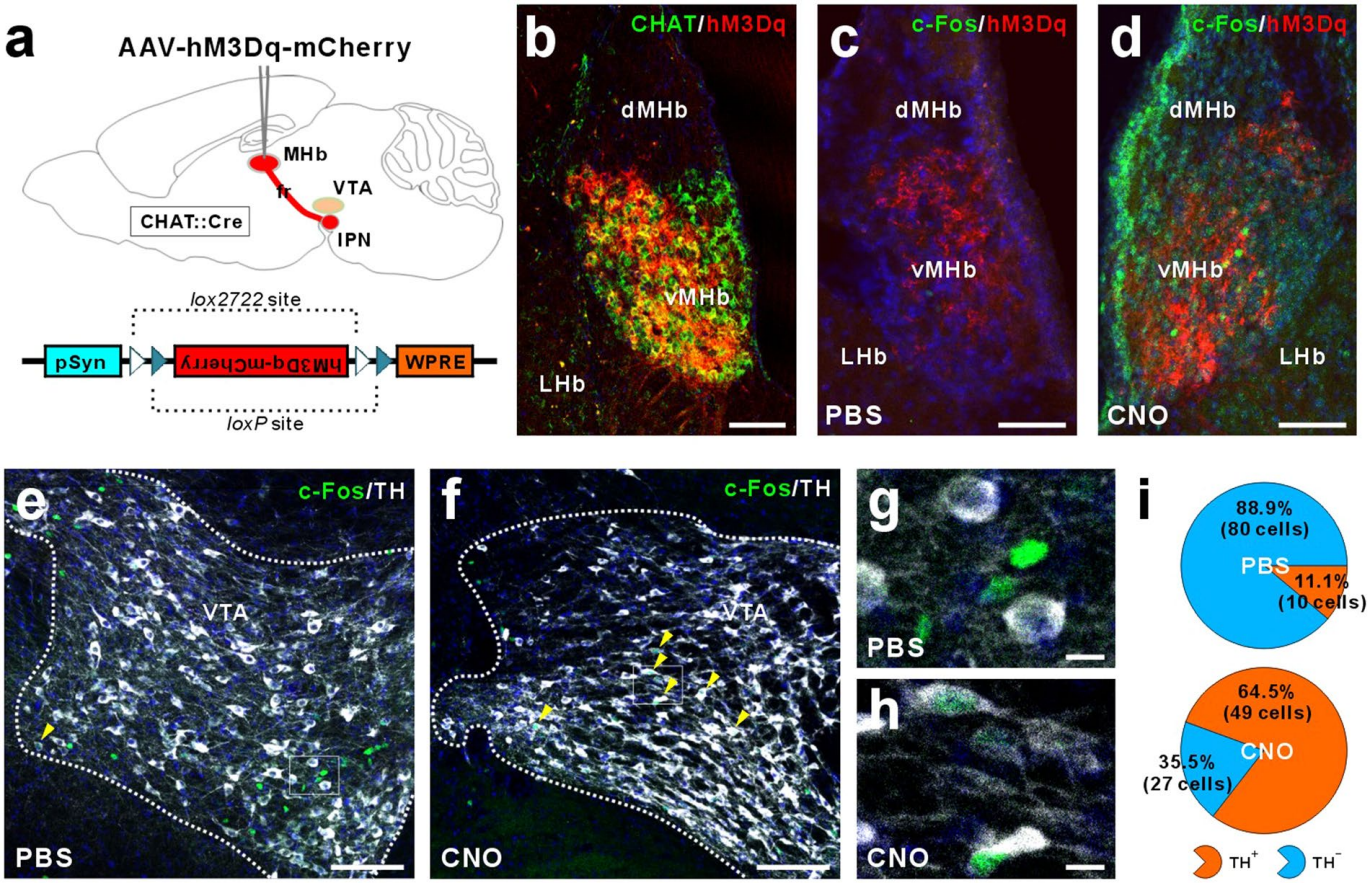
*In vivo* activation of MHb cholinergic neurons induces the excitation of dopamine neurons in the ventral tegmental area. (**a**) Pharmacogenetic stimulation by DREADD mice. We injected ChAT::cre mice with Cre-dependent hM3Dq virus (AAV-hM3Dq-mCherry) after 1 week habituation. (**b**) The mCherry-fused hM3Dq protein was expressed in the CHAT-positive habenula cholinergic neurons. (**c** and **d**) c-Fos expression (green) was induced in habenula cholinergic neurons expressing hM3Dq-mCherry (red) following pharmacogenetic selective stimulation for 60 min using i.p. injection of CNO. (**e**–**h**) CNO administration induced the increase of c-Fos immunoreactivity (green) in the TH-positive cells (i.e. dopamine neurons; white) and the decrease of c-Fos immunoreactivity in the TH-negative cells. Yellow arrow heads indicate the TH-positive and c-Fos-positive cells. (**i**) In CNO-injected DREADD mice, 64.5% of the dopamine neurons (n = 49 cells from 2 mice) and 35.5% of non-dopamine cells were c-Fos-positive. In contrast, saline-injected DREADD mice, 11.1% of the dopamine neurons (n = 10 cells from 2 mice) and 88.9% of non-dopamine cells were c-Fos-positive. Scale bar, (**b**–**f)** 100 μm; (**g** and **h)**, 10 μm.

**Figure 5:**
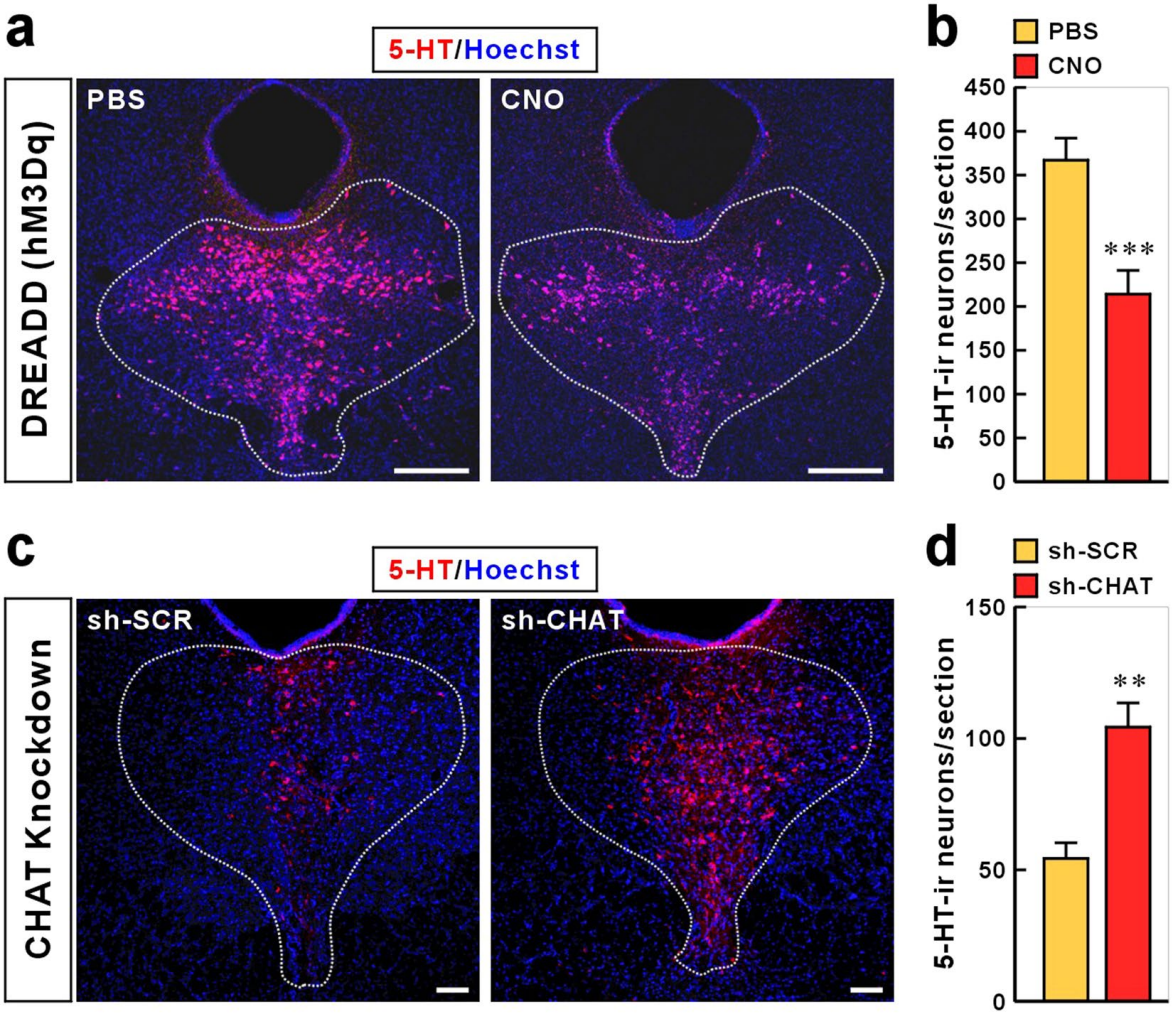
MHb cholinergic neurons regulate the population of 5-HT-immunoreactive neurons in the dorsal raphe nucleus. (**a** and **b**) DREADD mice expressing Cre-dependent hM3Dq were pharmacogenetically stimulated for 60 min using i.p. injection of CNO. Cell nuclei and 5-HT were visualized using Hoechst staining (blue) and Cy3 (red), respectively. CNO administration reduced the number of 5-HT-immunoreactive neurons in the DRN. Data represent mean ± SEM (PBS, n = 7 mice; CNO, n = 8; ***P < 0.001, Student’s *t*-test). Scale bar, 50 μm. (**c** and **d**) CHAT knockdown effect on the number of 5-HT-immunoreactive neurons in the DRN. Immunohistochemical analysis was performed using rats that were used for behavioral tests in Fig. 2. Cell nuclei and 5-HT were visualized using Hoechst staining (blue) and Cy3 (red), respectively. CHAT knockdown increased the number of 5-HT-immunoreactive neurons in the DRN Data represent mean ± SEM (sh-SCR, n = 5 rats; sh-CHAT, n = 5; **P < 0.01, Student’s *t*-test). Scale bar, 100 μm.

Since the VTA dopamine neurons modulate the motivation or hedonic state^25–27^, we investigated whether the selective activation of habenular cholinergic neurons could have a causal role in excitation of VTA dopamine neurons. We expressed the stimulatory DREADD, designated as “hM3Dq”, in the ChAT::cre mice (Fig. 4a–c). The DREADD was fused to mCherry and receptor expression was detected exclusively in the habenular CHAT neurons (Fig. 4b). A single intraperitoneal (i.p.) injection of CNO (5 mg/kg) into DREADD mouse induced the expression of immediate early gene c-Fos in DREADD overexpressed neurons of the MHb (Fig. 4d), suggesting that CNO-induced activation of DREADDing neurons was limited in the MHb cholinergic neuronal population. Altered activity of VTA neurons after CNO-induced MHb cholinergic stimulation was quantified by assaying the proportion of TH-immunopositive and TH-immunonegative cells that expressed the activity-dependent c-Fos. Strikingly, CNO significantly increased c-Fos immunoreactivity in the VTA dopamine neurons and decreased c-Fos immunoreactivity of non-dopamine cells (Fig. 4e–i). In addition, CNO-induced MHb cholinergic activation led to reduced number of 5-HT-immunoreactive (ir) neurons (and the intensity of somatic 5-HT-ir neurons) in the DRN (Fig. 5a and b). In contrast to this finding in hM3Dq mice, we found that CHAT knockdown in rat MHb increased the number of 5-HT-ir neurons in the DRN compared with control rats (Fig. 5c and d). These results suggest a possibility that MHb cholinergic neurons regulate VTA dopamine neurons and DRN serotonin neurons.

### Cholinergic Genes are Down-regulated in the Habenula of Depressed Suicide Victims

**Figure 6:**
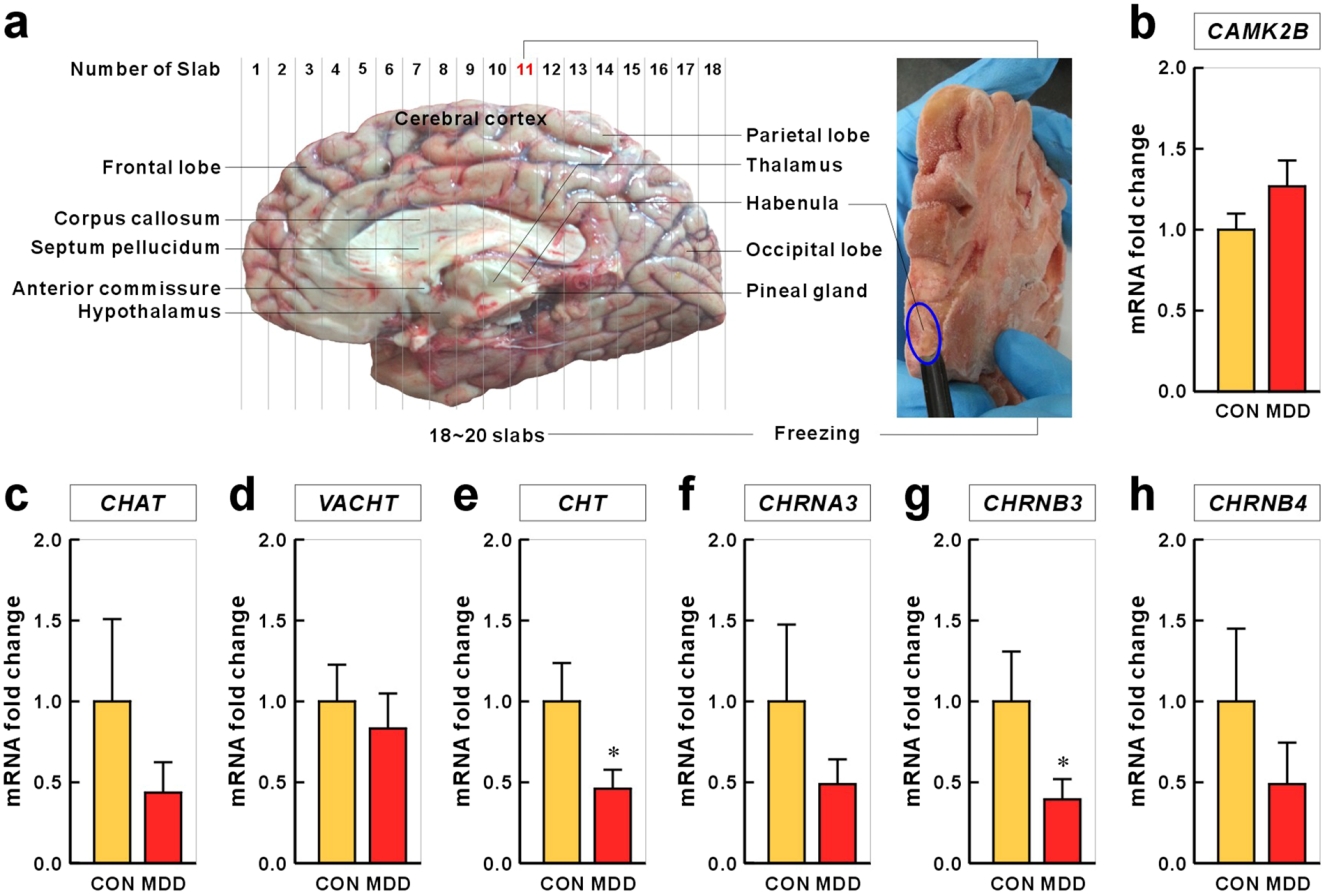
Expression of cholinergic system genes in habenula tissue obtained from persons with MDD who died by suicide. (**a**) The human brain was separated into two hemispheres by slicing through the center of the brain stem and cerebellum. Each hemisphere was sliced coronally into 18–20 slabs (slab 1, frontal lobe; slab 18, occipital lobe) and these slabs were frozen. The habenula is nearly adjacent to the pineal gland and is contained in the 11th of 18 slabs. To isolate habenula samples, a carving tool with a round tip was used to make long grooves one at a time. (**c**–**i**) The mRNA expression levels of CHAT, VACHT, CHT, CHRNA3, CHRNB3, and CHRNB4 were evaluated using TaqMan probe-based qRT-PCR. All cholinergic system genes examined were down-regulated in suicide, but not the control CAMK2B gene (**b**), an excitatory neuronal marker. GAPDH and β-Actin were used as reference genes to normalize qRT-PCR data. Data represent mean ± SEM (CON, Control, n = 11; MDD, n = 12 subjects; *P < 0.05, Mann–Whitney *U*-test).

Finally, we examined whether the down-regulated habenular cholinergic gene expression found in animal models of depression was recapitulated in depressed human suicide victims. Therefore, we established human brain tissue processing as follows. We subdivided intact brains into 18–20 slabs and froze them. From the frozen brain slabs, we isolated samples of the habenula (Fig. 6a). We performed qRT-PCR analysis with RNA extracted from postmortem habenula tissue of 12 people with MDD who had died by suicide (Fig. 6b–h; age, 41.5 ± 4.02 years; gender, male; postmortem intervals (PMI), 37.36 ± 4.23 h; pH, 6.48 ± 0.09) and 11 normal controls (age, 44 ± 3.04 years; gender, male; PMI, 25.78 ± 4.52 h; pH, 6.67 ± 0.04; no significant difference between controls and MDD suicides) to identify mRNA changes in cholinergic signaling genes. The mRNA expression levels of CHT (fold change for controls vs. MDD suicides: 1.000 ± 0.237 vs. 0.460 ± 0.117, *P* = 0.025) and CHRNB3 (1.000 ± 0.308 vs. 0.394 ± 0.126, *P* = 0.046) were significantly reduced in suicides relative to those in controls. The mRNA expression levels of the other cholinergic genes, CHAT (1.000 ± 0.509 vs. 0.436 ± 0.188, *P* = 0.559), VACHT (1.000 ± 0.227 vs. 0.833 ± 0.216, *P* = 0.186), CHRNA3 (1.000 ± 0.475 vs. 0.488 ± 0.154, *P* = 0.442) and CHRNB4 (1.000 ± 0.450 vs. 0.489 ± 0.257, *P* = 0.207), were not significantly changed, although a tendency for decreased expression was observed in MDD suicides. The negative control, CAMK2B, remained unchanged (1.000 ± 0.099 vs. 1.268 ± 0.160, *P* = 0.282).

## Discussion

The results of this study demonstrated that habenular cholinergic gene expression was significantly reduced in two animal models of depression and in post-mortem tissue of depressed persons who died by suicide. Anhedonia (markedly diminished interest or pleasure) and depressed mood (e.g., feeling sad, empty, or hopeless) are two core features of MDD, and at least one of these features is required for a diagnosis of MDD^28^. Although they are key symptoms of the same syndrome (i.e., depression), anhedonia and despair are related to distinct endophenotypes^29,30^. Because symptoms of these two features do not occur concurrently in a considerable proportion of patients with MDD, anhedonia and depressed mood may be associated with different etiological mechanisms (i.e., involvement of different neurotransmitters, pathways, neuronal connections, etc.). Approximately 37% of the patients diagnosed with MDD are estimated to experience clinically significant anhedonia^31^, and anhedonia is considered to be a particularly difficult symptom to treat. Current clinical evidence suggests that standard antidepressants (e.g. selective serotonin reuptake inhibitors) do not attain remission in motivational and reward-processing deficits in the majority of depressed patients with anhedonia^32–35^. In the present study, habenula-specific CHAT knockdown in rats induced behavior suggestive of anhedonia but not despair (Fig. 2). In concordance with previous clinical studies, this anhedonia-like behavior was not ameliorated by chronic fluoxetine treatment (Fig. 3). Therefore, it is plausible that abnormal habenular cholinergic signaling may be a major pathophysiological pathway mediating anhedonia-like symptom. We suggest that an animal model with down-regulated MHb CHAT would provide a promising tool for studying the distinct mechanisms underlying anhedonia but excluding despair.

Examinations of the functional role of the habenula in depressive disorder have been focused on the hyperactivity of LHb neurons. Recent studies showed that these LHb neurons may selectively excite dopaminergic VTA neurons projecting to the medial prefrontal cortex that elicit aversion and cognitive impairments^26^. In animal model of depression, increased LHb neuronal activity drives the excitation of GABAergic neurons in the rostromedial tegmental nucleus, which reduces dopaminergic activity and is potentially involved in the pathophysiology of depression^36^. Although LHb neurons also connect with serotonergic raphe nuclei and this LHb–serotonin circuit seems to be important in psychiatric disorders, little is known about how the LHb–serotonin circuit might be associated with depression^36^. Previous preclinical studies in nearly all animal studies, including species from zebra fish up to higher primates, have already linked the MHb–interpeduncular nucleus (IPN) cholinergic pathway to anxiety, fear, and nicotine addiction^36–38^. In addition to contributing to these mood disorders, because down-regulated MHb cholinergic signaling is involved in anhedonia-like behavior, the MHb–IPN cholinergic circuit represents an alternative pathway to the LHb–VTA circuit for regulating hedonic state.

The current theory of how the cholinergic system affects depression suggests that cholinergic hyperactivity leads to depression symptoms^39,40^. This hypothesis is supported by the clinical and preclinical findings that scopolamine, a non-selective muscarinic acetylcholine receptor antagonist, reverses depression-like behaviors or symptoms in an established animal model or in human patients^41,42^, and shRNA-mediated knockdown of acetylcholinesterase in mouse hippocampus induces the symptoms of depression^6^. In contrast to this general hypothesis, specific inactivation of the cholinergic interneurons of the nucleus accumbens causes depression-like behaviors, including anhedonia and behavioral despair^43^, and selective activation of accumbal cholinergic interneurons enhances accumbal extracellular dopamine release^44^, suggesting that cholinergic signaling may exert differential effects on mood depending on the brain region. Therefore, the cholinergic pathway of the MHb–IPN and the accumbal cholinergic interneurons belong to a parallel system, as reduced cholinergic neurotransmission in these systems induces depression-like phenotypes, which is in contrast to the hyperactivity theory of other cholinergic signaling in depression. However, previous studies found no direct evidence for a causal relation between cholinergic dysfunction and depression symptoms in human patients^8^. In the present study, we provided the first evidence that cholinergic signaling is down-regulated in the habenula of patients with MDD and that this reduced cholinergic activity may be associated with symptom of anhedonia.

According to a report by Aizawa *et al*., the habenula exhibits heterogeneity with respect to the differential expression of a combination of neurotransmitter marker genes, monoaminergic receptors, and previously unknown genes^45^. The CHAT-expressing region of the MHb is known to project axons to the central and intermediate subnuclei of the IPN^46^. When considering the neural circuitry that may underlie these behavioral effects, it is important to note that habenular cholinergic neurons stimulate IPN neurons by co-releasing glutamate and ACh^47^. Moreover, brief optogenetic stimulation of habenular cholinergic neurons produces fast excitatory postsynaptic currents that are mediated by glutamate receptor channels, whereas strong and sustained stimulation evokes slow inward currents that are largely mediated by nAChRs^47^. Therefore, the aversive stimuli-induced alteration of cholinergic activity in a specific brain region could be involved in pro-depressive or adaptive behaviors. Exposure to an acute stress may lead to adaptive behavioral responses mediated through ACh release in the medial habenulo-interpeduncular synapses, whereas chronic stress may lead to decreases in ACh signaling, resulting in anhedonia-like symptom. CRS-induced apathy-like phenotypes are ameliorated by facilitating ACh signaling through pharmacological inhibition of acetylcholinesterase^48^. Together with the down-regulation of genes associated with the cholinergic system in the MHb that we observed in both human suicides and rats subjected to a CRS or LH animal model of depression, this evidence suggests that decreased cholinergic activity in the MHb may cause anhedonia-like behavior.

How might MHb cholinergic signaling contribute to anhedonia-like symptom? Because dopamine neurons of the VTA strongly modulate hedonic behaviors^25,26^ and enhanced activity of MHb cholinergic neurons leads to increased activity of VTA dopamine neurons (Fig. 4), MHb cholinergic signaling may be associated with VTA dopamine neuron-dependent anhedonia-like behavior. How might the MHb–IPN cholinergic circuitry interconnect with VTA dopamine neurons? Since most MHb efferent neurons project to the IPN in the midbrain^46^, dopaminergic neurons in the VTA are thought to be regulated indirectly by MHb cholinergic neurons. Cholinergic projection neurons in the MHb form synapses in the IPN, where cholinergic and glutamatergic co-transmission from the MHb neurons to the IPN is essential for function and behavior. Presynaptic nAChRs on MHb neurons are important for facilitating glutamate release in MHb–IPN terminals^49,50^. Conditional elimination of ACh in MHb neurons reduces glutamate reuptake in synaptic vesicles and abolishes nAChR-mediated presynaptic facilitation of glutamate release^51^. The down-regulation of MHb cholinergic nAChRs as well as CHAT in a CRS animal model of depression and in humans with MDD (Figs 1 and 6) is more likely to induce profound deficits in MHb–IPN synaptic transmission. The IPN innervates and has reciprocal connections with the dorsal raphe (DRN) and median raphe (MRN) nuclei, which are involved in the release of serotonin throughout the brain^52–54^. A recent optogenetic study reported that neurons in the DRN encode reward processing through their release of serotonin and glutamate at VTA dopamine neurons^55^. Consistent with this neural circuit structure, our results showed that activation of MHb cholinergic neurons (DREADD mice; Fig. 5a and b) reduced the number of 5-HT-ir neurons and *vice versa* (CHAT knockdown in rats; Fig. 5c and d). The reduced number of 5-HT-ir neurons in the DRN may be associated with extracellular serotonin levels. The number and density of serotonergic neurons were also found to be higher in suicide victims than in controls^56^. The IPN also has reciprocal connections with other midbrain areas, such as the lateral dorsal tegmental nucleus (LDTg) or the periaqueductal gray, which may play important roles in the expression of emotional behavior^54,57^. Optogenetic excitation of the LDTg axons in the VTA reinforces appetitively motivated behaviors^58^. In addition, selective photo-excitation of VTA-projecting cholinergic terminals from the LDTg causes reward reinforcement^59^. The MHb–IPN cholinergic pathway may regulate VTA activity via its projections to the LDTg. Therefore, because the functions of IPN efferents and afferents remains very poorly characterized, further study is warranted to determine which downstream neural circuits of habenular cholinergic neurons are responsible for anhedonia-like behavior.

## Methods

### Animals and Behavioral Paradigms

All experimental procedures with animals were approved by Korea University Institutional Animal Care and Use Committee and performed in accordance with the guidelines of Korea University. Sprague-Dawley male rats (purchased from Japan SLC, Inc. Shizuoka, Japan) were used. The procedure of chronic restraint stress induction protocol was performed as described previously^60^. The animals were habituated for one week before experimentation. Animals were randomly assigned to either of two groups: an experimental group (chronic restraint stress, CRS), in which the animals were exposed to restraint stress for two hours daily for two weeks or a control group (non-stressed, NS), in which the animals remained in their home cage and handled for 5 min per day. During restraint, rats were placed into a breathable decapicone (DC 200, DecapiCones, Braintree Scientific, Braintree, MA, USA). Body weight was monitored throughout the experimental period. On the last day of the stress period, rats were sacrificed immediately after the last stress exposure. Animal behavioral tests were performed including the sucrose preference test (SPT) and the forced swim test (FST). For generation of learned helpless rats, we used the paradigm based on previously published procedures^15^. Briefly, rats were exposed to learned helplessness training session. This training session consisted of 120 inescapable, uncontrollable electric foot shocks delivered at an intensity of 0.8 mA for a duration of 20 s and an unpredictable inter-shock interval of 5–15 s in shock chambers (chamber dimensions, 30 cm wide × 30 cm deep × 25 cm high; TSE, Bad Homburg, Germany). Control rats were placed in the shock chamber for the same period of time without being shocked. Twenty-four hours after the third training session, learned helplessness was assessed using the shuttle-box active avoidance task. Rats were placed on one side of a shuttle box that was separated into two equal compartments by an archway and allowed to explore the shuttle box for 5 min. The helplessness response was evaluated by 30 trials of unpredictable but escapable foot shock delivered at an intensity of 1.2 mA for a maximum duration of 10 s and an unpredictable inter-shock interval of 12–36 s. The foot shock was terminated immediately when the mouse had completely crossed to the other side of the box. LH was determined by escape failures and escape latencies. An escape failure was recorded when the rat failed to cross to the other side to terminate the shock. The escape latency was the amount of time the rat took to move to the other side.

### Radioimmunoassay for Plasma Corticosterone

The effect of CRS on circulating corticosterone levels was measured as follows. Trunk blood was collected immediately after decapitation, centrifuged (10,000 × *g*, 10 min, 4 °C). Following plasma separation, circulating levels of corticosterone were measured by radioimmunoassay using a Coat-a-Count Rat Corticosterone kit (TKRC1, Siemens Healthcare Diagnostics, Los Angeles, CA, USA).

### Animal Behavioral Paradigms

For the sucrose preference test (SPT), all rat were singly housed in individual cages. Prior to beginning testing, rats were habituated to the presence of two drinking bottles (one with 1% sucrose and the other with tap water) for 2 days in their home cage. The positions of two bottles were switched daily to reduce any side preference. On the day of the test, rats were water deprived for 12 h and then were exposed to two identical bottles (one with 1% sucrose and the other with tap water) for 1 h. Sucrose and water consumption were recorded by re-weighing the pre-weighed bottles of test solutions. Sucrose preference was calculated as a relative ratio (mass of sucrose solution intake/total fluid intake). For the forced swim test (FST), rats were exposed to a pre-swim 24 h prior to test for 15 min in a clear cylinder filled with 24–25 °C water. The test sessions were performed 24 h later and animal behavior was videotaped for 5 min using infrared camera. The cylinder was 45 cm diameter and 60 cm high. The depth of the water was set to allow measurement of active (swimming and climbing) or passive (immobility) behaviors without touching the bottom with their hind limbs.

### Rat Brain Samples and qRT-PCR

Habenula was isolated from the brain immediately after decapitation in RNA*later* RNA Stabilization Solution (AM7020, Ambion, Austin, TX, USA). Total RNA was extracted using TRIzol reagent (Invitrogen, Carlsbad, CA, USA). The total RNA sample (1 μg) was reverse-transcribed by reverse-transcriptase (Promega, Madison, WI, USA) and oligo (dT) primer. qRT-PCR was performed with 0.25 μg of RT product in the presence of specific primer sets (Supplementary Table S1) and the Thermo Scientific Maxima SYBR Green qPCR Master Mix (2×) (#K0251, Fermentas, MARYLAND, USA) using the LightCycler 1.5 Instrument (Roche, Basel, Switzerland). Final products of qPCR were electrophoresed on 2% agarose gels and visualized by staining with SafeView Nucleic Acid Stain (G108, Applied Biological Materials, Canada). The cycle numbers (C_t_) of the critical point at which the fluorescent signal exceeded the background for each gene were determined by qRT-PCR and expression values for each gene were subsequently normalized to expression values of GAPDH as an endogenous control within each sample. Relative quantification used to calculate the fold change between CRS and NS groups was performed using the comparative C_t_ method (ΔΔC_t_).

### RNAscope Assay

*In situ* hybridization histochemistry was performed basically as described by Stempel *et al*.^61^. In brief, frozen sections (14 µm thick) were cut coronally through the habenula formation. Sections were thaw-mounted onto Superfrost Plus Microscope Slides (Fisher Scientific, Waltham, USA), fixed in 4% formaldehyde for 10 min, dehydrated in increasing concentrations of ethanol for 5 min, and finally air-dried. Tissues were then pretreated for protease digestion for 10 min at room temperature. For RNA detection, incubations with the different amplifier solutions were performed in a HybEZ hybridization oven (Advanced Cell Diagnostics, Hayward, CA) at 40 °C. The labeled probes were conjugated to Atto 550. The sections were hybridized for 2 h at 40 °C with labeled probe mixture per slide. The nonspecifically hybridized probe was removed by washing the sections, three times for 2 min each in 1× wash buffer at room temperature, followed by Amplifier 1-FL for 30 min, Amplifier 2-FL for 15 min, Amplifier 3-FL for 30 min, and Amplifier 4 Alt B-FL for 15 min at 40 °C. Each amplifier was removed by washing with 1× wash buffer for 2 min at room temperature. The slides were viewed, analyzed, and photographed with an LSM 700 microscope (Zeiss). Image analysis was performed in image J program. The sequences for probe generation were as follows: CHAT, 259–1141 of NM_001170593.1 (NCBI Gene accession number); VACHT, 1693–2837 of NM_031663.2; CHT, 123–998 of NM_053521.1; CHRNA3, 984–2228 of NM_052805.2; CHRNB3, 255–1336 of NM_133597.1; CHRNB4, 1139–2433 of NM_052806.2; CAMK2B, 1439–3560 of NM_001042354.1 (Advanced Cell Diagnostics, Hayward, CA).

### Viral Vector and Administration into Brain

The AAV-CHAT-RNAi (RNA interference) was constructed using pAAV-U6-GFP vector (provided by Cell Biolabs, Inc., San Diego, USA), which contains a PGK promoter expressing EGFP and a U6 promoter expressing shRNA. The sequence of CHAT shRNA is 5′-GAGCGAGCCTTGTTGACAT-3′. The sequence of a scrambled RNAi used as a control is 5′-TCGTCATAGCGTGCATAGG-3′^62^. The AAV virus packaging was supported by the Penn Vector Core at the University of Pennsylvania. For viral injection, 8 weeks-old SD rats were anesthetized with pentobarbital (50 mg/kg of body weight) by i.p. injection and placed in a stereotaxic apparatus. Rats were injected bilaterally with 1 μl of concentrated AAV virus into the MHb (coordinates from bregma: −3.0 mm anterior/posterior, ± 2.75 mm medial/lateral, −5.28 mm dorsal/ventral, with 26° angle toward the midline in the coronal plane) using microinjection cannula (30 gauge, Plastics One, Roanoke, USA) at a slow rate (50 nl/min). The injection cannula was slowly withdrawn 20 min after virus infusion. Behavior experiments were performed at least 21 days after surgery. The injection sites were examined at the end of all the behavior tests and only data from animals with correct injections were included.

### Immunohistochemistry and Western Blot

For verification of AAV-mediated CHAT silencing in habenula, coronal rat brain sections (30 μm) permeabilized by 0.3% Triton X-100 in PBS were incubated with primary antibodies against CHAT (1:100, Abcam), EGFP (1:500, Abcam), VACHT (1:500, Synaptic Systems), and 5-HT (1:2000, ImmunoStar) followed by Cy3- and Alexa 488-conjugated secondary antibodies (Jackson ImmunoResearch). The nucleus was visualized using Hoechst (1:1000, Invitrogen). Fluorescent images were acquired using a confocal microscope (LSM710). For western blot, 15 μg habenular proteins were immunoblotted with the following antibodies; goat anti-CHAT (1:1000, Abcam), mouse anti-β-actin (1:5000, Sigma-Aldrich), and rabbit anti-VACHT (1:1000, Synaptic Systems).

### Drug administration

Fluoxetine was purchased from Sigma-Aldrich. Animals were administrated with injectable fluoxetine solution (diluted in phosphate buffered saline, PBS; 135 mM NaCl, 2.7 mM KCl, 4.3 mM Na_2_HPO_4_, 1.4 mM KH_2_PO_4_, pH 7.4) once a day over three-week period. For experiment to determine fluoxetine effect on CHAT knockdown-induced anhedonia-like behavior, rats were injected i.p. with saline or fluoxetine (10 mg/kg).

### DREADD mice

To validate whether the habenular cholinergic neurons regulate the activity of dopamine neurons in the VTA, we used Gq-coupled DREADD (designer receptors exclusively activated by designer drug) to stimulate the MHb cholinergic neurons. For this the ChAT::cre mice were infected with 1 μl of AAV-DIO-hSyn-hM3Dq-mCherry virus bilaterally into the MHb with coordinating from bregma: −3.14 mm anterior/posterior, ±0.83 mm medial/lateral, −3.05 mm dorsal/ventral, with 10° angle toward the midline in the coronal plane. Four weeks later, ChAT::cre mice virally expressing hM_3_Dq received an i.p. injection of PBS (control) or clozapine N-oxide (CNO; 5 mg/kg; Enzo Life Sciences, NY, USA).

### Human subjects

The Douglas Bell Canada Brain Bank (DBCBB; www.douglasbrainbank.ca; Douglas Mental Health University Institute, Verdun, Quebec) is a brain bank that recruits suicide cases and sudden death control subjects. To avoid prolonged agonal states, both cases and controls recruited to the bank cannot undergo resuscitation procedures or medical intervention. Brains are collected after consent is obtained from next-of-kin. To obtain diagnostic information, families were re-contacted a few months following recruitment to undergo a series of structured interviews with the person best acquainted with the deceased, a process commonly known as psychological autopsy. Interviews were supplemented with medical charts or other records, such as files from police, coroner or social services. Following the interviews, clinical vignettes were produced and assayed by a panel of clinicians to generate DSM-IV diagnostic criteria. In addition, samples from brain tissue, peripheral blood and urine were used for toxicological analysis. Once enrolled, brains were sectioned, flash frozen in isopentane, and then stored at −80 °C by experienced histopathologists.

### Human Habenula Dissection

For this study, we obtained tissue from the habenula from 12 suicide subjects diagnosed with major depression and 11 psychiatrically healthy control subjects. All subjects were male Caucasians and groups (suicides and controls) were matched for age, pH, and postmortem intervals (PMI). Habenula was carefully dissected as follows: human brain was divided into left and right hemispheres with cutting center of the brain stem and cerebellum and the meninges of the hemispheres were carefully removed. The trunk from the hemisphere was separated with a scalpel between the mammillary body and superior colliculus. Sample pH was measured using the cerebellum. To make slabs, the brain hemispheres was placed with the median face down on the cutting plate, cut coronally into 18–20 pieces and then frozen. Since the habenula typically exists in the 11th or 12th slabs, the habenula was gently removed from the frozen slab using a burin with round-shaped tip.

### Expression Analysis of Human Habenular Cholinergic Genes

To evaluate the cholinergic gene expression using human brain sample, TaqMan quantitative real-time polymerase chain reaction (qRT-PCR) analysis was performed. The total RNA sample (500 ng) was reverse-transcribed by reverse transcriptase (Invitrogen, Carlsbad, CA, USA) and random primer, and RNA integrity was determined with an Agilent 2100 Bioanalyzer (Agilent Technologies, Palo Alto, CA, USA). The stability of 28S ribosomal DNA was qualified with PCR method and not changed between control and suicide subjects (expression stability of 28S rDNA: control, 6.123 ± 0.1487; suicide, 6.154 ± 0.0728; Mann–Whitney *U*-test, *P* = 0.4235). Validation of housekeeping reference genes, such as GAPDH, TBP and CYC1, showed no differences between groups (Supplementary Table S2). qRT-PCR reactions were performed in triplicate with 2 μl of cDNA, 1 μl of TaqMan Expression Assay specific to each quantified gene and 10 μl of TaqMan Mastermix (Applied Biosystems, Foster City, CA, USA) according to manufacturer’s protocol using the LightCycler 1.5 Instrument (Roche, Basel, Switzerland). A standard curve was made of a pool of cDNAs from all subjects. Expression levels were calculated using the Absolute Quantification standard curve method with GAPDH. The thermal cycling conditions were set as follows: 95 °C for 5 min, 50 cycles each of 95 °C for 10 sec and 60 °C for 30 sec. The TaqMan probes are summarized in Supplementary Table S3.

### Statistical analysis

Statistical analyses were conducted using SPSS v. 20 for Windows (IBM corp., Armonk, NY, USA). If a data set was well modeled by a normal distribution, Student’s *t*-test was applied. A data set which is not normally distributed was conducted with the non-parametric Mann–Whitney *U*-test. *P* < 0.05 was considered significant. Values are expressed as mean ± SEM.

## Electronic supplementary material

Supplementary Information

## References

[1] Ferrari AJ (2013). Burden of depressive disorders by country, sex, age, and year: findings from the global burden of disease study 2010. PLoS Med.

[2] Dilsaver SC (1986). Cholinergic mechanisms in depression. Brain research.

[3] Janowsky DS, el-Yousef MK, Davis JM, Sekerke HJ (1972). A cholinergic-adrenergic hypothesis of mania and depression. Lancet.

[4] Janowsky DS, Risch SC, Gillin JC (1983). Adrenergic-cholinergic balance and the treatment of affective disorders. Prog Neuropsychopharmacol Biol Psychiatry.

[5] Risch SC, Cohen RM, Janowsky DS, Kalin NH, Murphy DL (1980). Mood and behavioral effects of physostigmine on humans are accompanied by elevations in plasma beta-endorphin and cortisol. Science.

[6] Mineur YS (2013). Cholinergic signaling in the hippocampus regulates social stress resilience and anxiety- and depression-like behavior. Proceedings of the National Academy of Sciences of the United States of America.

[7] Shytle RD (2002). Nicotinic acetylcholine receptors as targets for antidepressants. Mol Psychiatry.

[8] Drevets WC, Zarate CA, Furey ML (2013). Antidepressant effects of the muscarinic cholinergic receptor antagonist scopolamine: a review. Biological psychiatry.

[9] Klemm WR (2004). Habenular and interpeduncularis nuclei: shared components in multiple-function networks. Med Sci Monit.

[10] Lecourtier L, Kelly PH (2007). A conductor hidden in the orchestra? Role of the habenular complex in monoamine transmission and cognition. Neurosci. Biobehav. Rev..

[11] Hsu YW (2013). Medial habenula output circuit mediated by alpha5 nicotinic receptor-expressing GABAergic neurons in the interpeduncular nucleus. The Journal of neuroscience: the official journal of the Society for Neuroscience.

[12] Zhao-Shea R, Liu L, Pang X, Gardner PD, Tapper AR (2013). Activation of GABAergic neurons in the interpeduncular nucleus triggers physical nicotine withdrawal symptoms. Curr Biol.

[13] Dao DQ, Perez EE, Teng Y, Dani JA, De Biasi M (2014). Nicotine enhances excitability of medial habenular neurons via facilitation of neurokinin signaling. The Journal of neuroscience: the official journal of the Society for Neuroscience.

[14] Sartorius A, Henn FA (2007). Deep brain stimulation of the lateral habenula in treatment resistant major depression. Med. Hypotheses.

[15] Li B (2011). Synaptic potentiation onto habenula neurons in the learned helplessness model of depression. Nature.

[16] Renier N (2014). iDISCO: a simple, rapid method to immunolabel large tissue samples for volume imaging. Cell.

[17] Prado VF, Roy A, Kolisnyk B, Gros R, Prado MA (2013). Regulation of cholinergic activity by the vesicular acetylcholine transporter. Biochem J.

[18] Grady SR (2001). Nicotinic agonists stimulate acetylcholine release from mouse interpeduncular nucleus: a function mediated by a different nAChR than dopamine release from striatum. J Neurochem.

[19] Liang SD, Vizi ES (1997). Positive feedback modulation of acetylcholine release from isolated rat superior cervical ganglion. J Pharmacol Exp Ther.

[20] Gotti C (2009). Structural and functional diversity of native brain neuronal nicotinic receptors. Biochem Pharmacol.

[21] Grady SR (2009). Rodent habenulo-interpeduncular pathway expresses a large variety of uncommon nAChR subtypes, but only the alpha3beta4* and alpha3beta3beta4* subtypes mediate acetylcholine release. The Journal of neuroscience: the official journal of the Society for Neuroscience.

[22] Misgeld T (2002). Roles of neurotransmitter in synapse formation: development of neuromuscular junctions lacking choline acetyltransferase. Neuron.

[23] Erickson JD (1994). Functional identification of a vesicular acetylcholine transporter and its expression from a “cholinergic” gene locus. J Biol Chem.

[24] Picciotto MR, Higley MJ, Mineur YS (2012). Acetylcholine as a neuromodulator: cholinergic signaling shapes nervous system function and behavior. Neuron.

[25] Tye KM (2013). Dopamine neurons modulate neural encoding and expression of depression-related behaviour. Nature.

[26] Lammel S (2012). Input-specific control of reward and aversion in the ventral tegmental area. Nature.

[27] Koob GF (1996). Hedonic valence, dopamine and motivation. Mol Psychiatry.

[28] American Psychiatric Association. *Desk reference to the diagnostic criteria from DSM-5*. (American Psychiatric Publishing, 2013).

[29] Zimmerman M, McGlinchey JB, Young D, Chelminski I (2006). Diagnosing major depressive disorder: II: is there justification for compound symptom criteria?. J Nerv Ment Dis.

[30] Wacker J, Dillon DG, Pizzagalli DA (2009). The role of the nucleus accumbens and rostral anterior cingulate cortex in anhedonia: integration of resting EEG, fMRI, and volumetric techniques. Neuroimage.

[31] Pelizza L, Ferrari A (2009). Anhedonia in schizophrenia and major depression: state or trait?. Ann Gen Psychiatry.

[32] Dunlop BW, Nemeroff CB (2007). The role of dopamine in the pathophysiology of depression. Arch Gen Psychiatry.

[33] McCabe C, Cowen PJ, Harmer CJ (2009). Neural representation of reward in recovered depressed patients. Psychopharmacology (Berl).

[34] McCabe C, Mishor Z, Cowen PJ, Harmer CJ (2010). Diminished neural processing of aversive and rewarding stimuli during selective serotonin reuptake inhibitor treatment. Biological psychiatry.

[35] Price J, Cole V, Goodwin GM (2009). Emotional side-effects of selective serotonin reuptake inhibitors: qualitative study. Br J Psychiatry.

[36] Proulx CD, Hikosaka O, Malinow R (2014). Reward processing by the lateral habenula in normal and depressive behaviors. Nature neuroscience.

[37] Kobayashi Y (2013). Genetic dissection of medial habenula-interpeduncular nucleus pathway function in mice. Frontiers in behavioral neuroscience.

[38] Agetsuma M (2010). The habenula is crucial for experience-dependent modification of fear responses in zebrafish. Nature neuroscience.

[39] Saricicek A (2012). Persistent beta2*-nicotinic acetylcholinergic receptor dysfunction in major depressive disorder. Am J Psychiatry.

[40] Bell KA, Shim H, Chen CK, McQuiston AR (2011). Nicotinic excitatory postsynaptic potentials in hippocampal CA1 interneurons are predominantly mediated by nicotinic receptors that contain alpha4 and beta2 subunits. Neuropharmacology.

[41] Drevets WC, Furey ML (2010). Replication of scopolamine’s antidepressant efficacy in major depressive disorder: a randomized, placebo-controlled clinical trial. Biological psychiatry.

[42] Navarria A (2015). Rapid antidepressant actions of scopolamine: Role of medial prefrontal cortex and M1-subtype muscarinic acetylcholine receptors. Neurobiology of disease.

[43] Warner-Schmidt JL (2012). Cholinergic interneurons in the nucleus accumbens regulate depression-like behavior. Proceedings of the National Academy of Sciences of the United States of America.

[44] Cachope R (2012). Selective activation of cholinergic interneurons enhances accumbal phasic dopamine release: setting the tone for reward processing. Cell reports.

[45] Aizawa H, Kobayashi M, Tanaka S, Fukai T, Okamoto H (2012). Molecular characterization of the subnuclei in rat habenula. The Journal of comparative neurology.

[46] Andres KH, von During M, Veh RW (1999). Subnuclear organization of the rat habenular complexes. The Journal of comparative neurology.

[47] Ren J (2011). Habenula “cholinergic” neurons co-release glutamate and acetylcholine and activate postsynaptic neurons via distinct transmission modes. Neuron.

[48] Martinowich K (2012). Acetylcholinesterase inhibition ameliorates deficits in motivational drive. Behav Brain Funct.

[49] Girod R, Barazangi N, McGehee D, Role LW (2000). Facilitation of glutamatergic neurotransmission by presynaptic nicotinic acetylcholine receptors. Neuropharmacology.

[50] Girod R, Role LW (2001). Long-lasting enhancement of glutamatergic synaptic transmission by acetylcholine contrasts with response adaptation after exposure to low-level nicotine. The Journal of neuroscience: the official journal of the Society for Neuroscience.

[51] Frahm S (2015). An essential role of acetylcholine-glutamate synergy at habenular synapses in nicotine dependence. eLife.

[52] Sutherland RJ (1982). The dorsal diencephalic conduction system: a review of the anatomy and functions of the habenular complex. Neurosci Biobehav Rev.

[53] Herkenham M, Nauta WJ (1979). Efferent connections of the habenular nuclei in the rat. The Journal of comparative neurology.

[54] Molas S, DeGroot SR, Zhao-Shea R, Tapper AR (2017). Anxiety and Nicotine Dependence: Emerging Role of the Habenulo-Interpeduncular Axis. Trends in pharmacological sciences.

[55] Liu Z (2014). Dorsal raphe neurons signal reward through 5-HT and glutamate. Neuron.

[56] Malberg JE, Duman RS (2003). Cell proliferation in adult hippocampus is decreased by inescapable stress: reversal by fluoxetine treatment. Neuropsychopharmacology.

[57] Groenewegen HJ, Ahlenius S, Haber SN, Kowall NW, Nauta WJ (1986). Cytoarchitecture, fiber connections, and some histochemical aspects of the interpeduncular nucleus in the rat. The Journal of comparative neurology.

[58] Steidl S, Veverka K (2015). Optogenetic excitation of LDTg axons in the VTA reinforces operant responding in rats. Brain research.

[59] Xiao C (2016). Cholinergic Mesopontine Signals Govern Locomotion and Reward through Dissociable Midbrain Pathways. Neuron.

[60] Andrus BM (2012). Gene expression patterns in the hippocampus and amygdala of endogenous depression and chronic stress models. Mol Psychiatry.

[61] Stempel AJ, Morgans CW, Stout JT, Appukuttan B (2014). Simultaneous visualization and cell-specific confirmation of RNA and protein in the mouse retina. Mol Vis.

[62] Santamaria J (2009). Silencing of choline acetyltransferase expression by lentivirus-mediated RNA interference in cultured cells and in the adult rodent brain. J Neurosci Res.

