# Author Correction: Down-regulation of cholinergic signaling in the habenula induces anhedonia-like behavior

**DOI:** 10.1038/s41598-017-16991-1

**Published:** 2017-12-01

**Authors:** Seungrie Han, Soo Hyun Yang, Jin Yong Kim, Seojung Mo, Esther Yang, Ki Myung Song, Byung-Joo Ham, Naguib Mechawar, Gustavo Turecki, Hyun Woo Lee, Hyun Kim

**Affiliations:** 1 Department of Anatomy, College of Medicine, Korea University, Seoul, 136-705 Korea; 2 Department of Psychiatry, College of Medicine, Korea University, Seoul, 136-705 Korea; 3 Department of Psychiatry, McGill University, Douglas Mental Health University Institute, Montreal, QC H4H 1R3 Canada

*Scientific Reports* **7**:900; doi:10.1038/s41598-017-01088-6; Article published online 18 April 2017

The Acknowledgements section in this Article is incomplete:

“We thank Professor Margaret DeAngelis (University of Utah, USA) and Dr. Hemin Chin (NIH, USA) for the critical comments on the manuscripts. This work was supported by the Brain Program through the National Research Foundation of Korea (NRF), funded by the Ministry of Science, ICT & Future Planning (NRF-2011-0019227, NRF-2013M3C7A1056732 and NRF-2012M3A9B6055378 to H. Kim; NRF-2011-0019229 and NRF-2013R1A1A2060527 to H.W. Lee), and the Brain Korea 21PLUS. Bioedit edited the manuscript”.

should read:

“We thank Professor Margaret DeAngelis (University of Utah, USA) and Dr. Hemin Chin (NIH, USA) for the critical comments on the manuscripts. This work was supported by the Brain Program through the National Research Foundation of Korea (NRF), funded by the Ministry of Science, ICT & Future Planning (NRF-2011-0019227, NRF-2013M3C7A1056732, NRF-2012M3A9B6055378, and NRF-2015M3C7A1029034 to H. Kim; NRF-2011-0019229 and NRF-2013R1A1A2060527 to H.W. Lee), and the Brain Korea 21PLUS. Bioedit edited the manuscript”.

