# Supplementary material for: Down-regulation of cholinergic signaling in the habenula induces anhedonia-like behavior
Source: Sci Rep. 2017 Apr 18;7:900. doi: 10.1038/s41598-017-01088-6 (PMC5429859; doi:10.1038/s41598-017-01088-6)
Supplement: Supplementary file 1 — Supplementary Information [file 41598_2017_1088_MOESM1_ESM.doc]

**SUPPLEMENTARY INFORMATION**

Down-regulation of cholinergic signaling in the habenula induces anhedonia-like behavior

Seungrie Han^1^**^†^**, Soo Hyun Yang^1^**^†^**, Jin Yong Kim^1^**^†^**, Seojung Mo^1^, Esther Yang^1^, Ki Myung Song^1^, Byung-Joo Ham^2^, Naguib Mechawar^3^, Gustavo Turecki^3^, Hyun Woo Lee^1^*, and Hyun Kim^1^*

Supplementary Figures


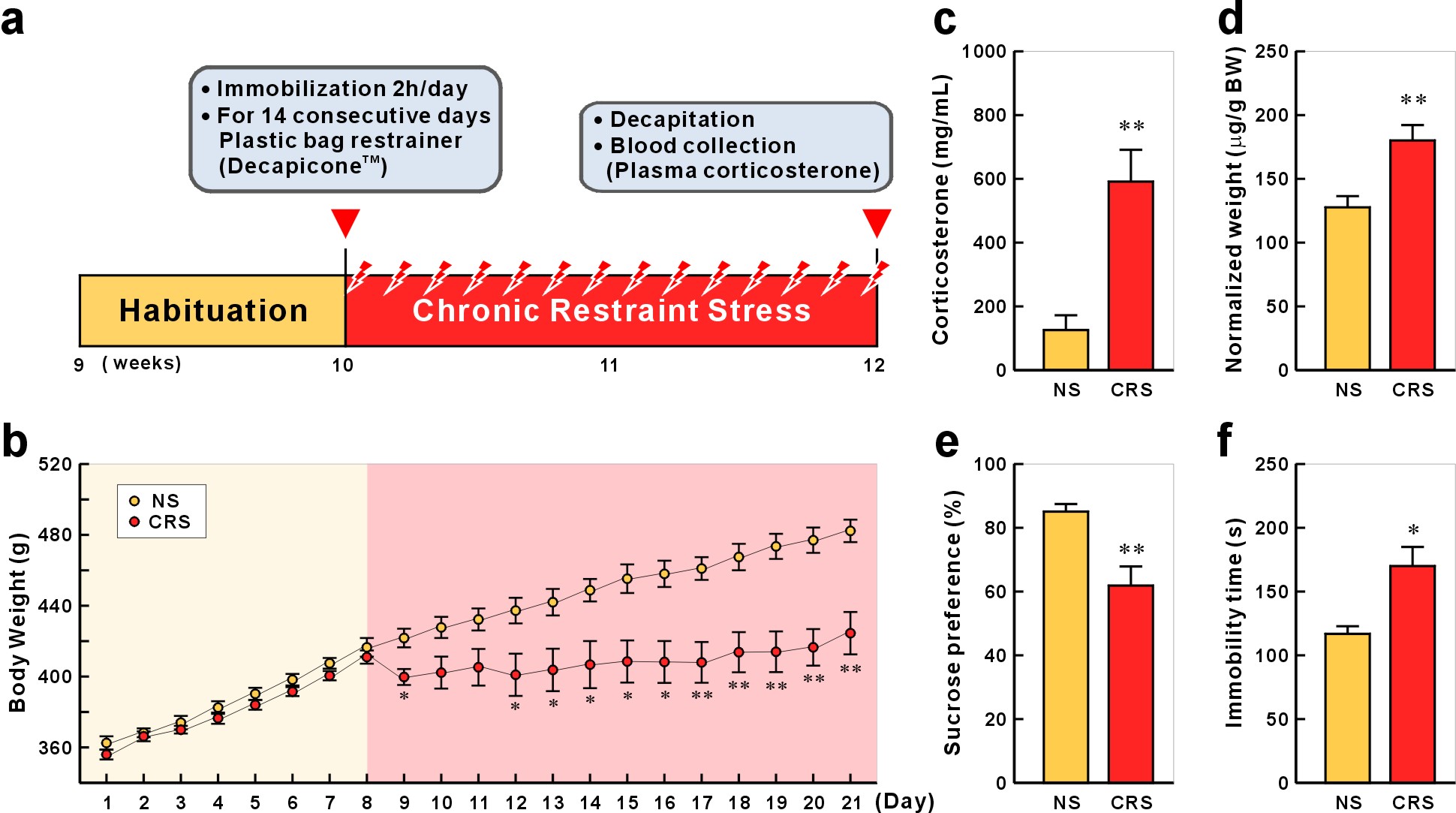


Figure S1. Generation of chronic restraint stress-induced animal model of depression

(**a**) Schematic diagram depicting the steps in the stress experiment and tissue sampling. (**b**) Body weight gain during the stress experiment. Rats were weighed daily before the stress procedure. CRS began on Day 8; NS, non-stressed, n = 4; CRS, n = 4. CRS increases plasma corticosterone levels (**c**) and induces adrenal gland enlargement (**d**). Adrenal gland weight is normalized to body weight. The data are shown as mean ± SEM. **P* < 0.05, ***P* < 0.01, Student’s *t*-test. CRS rats show depression-like behaviors, that is, anhedonia in the SPT (**e**) and depressed mood in the FST (**f**). Data represent mean ± SEM. NS, n = 20; CRS, n = 20 rats, ***P* < 0.01, Mann−Whitney *U*-test.


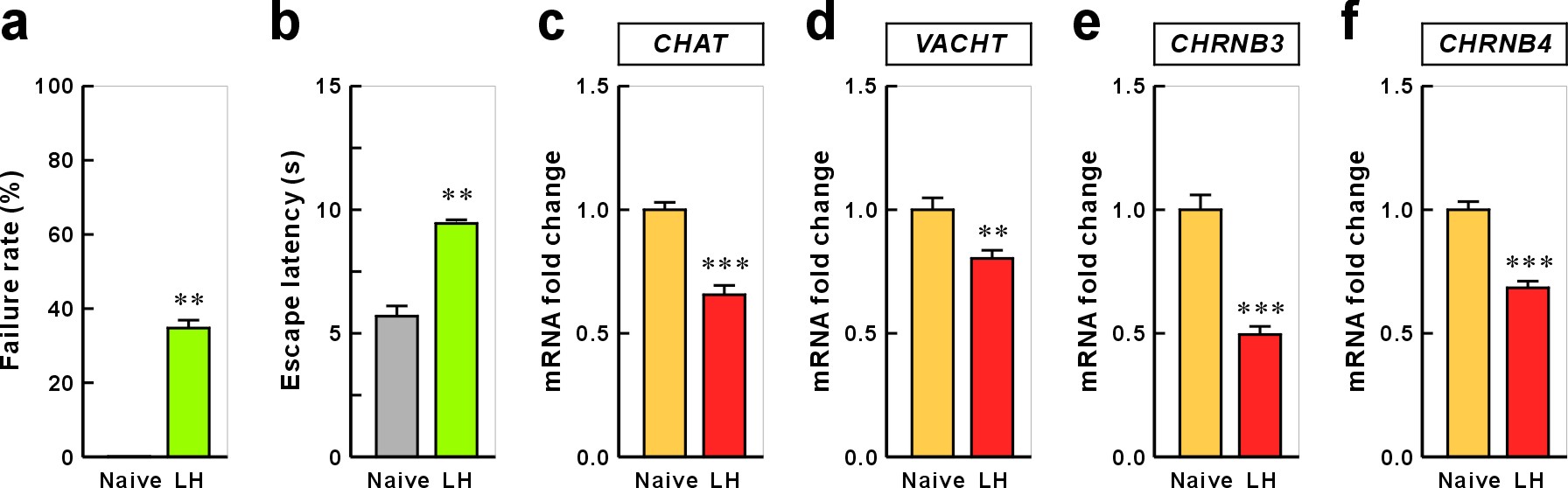


Figure S2. Cholinergic signaling genes in the habenula of learned helpless rats

Rats subjected to the learned helplessness procedure (see SI methods) exhibited increased failure rate (a) and escape latency time (b) in the active avoidance test. (c−f) Cholinergic signaling genes were down regulated in the habenula of learned helpless rats. Data represent mean ± SEM. Naïve, control animal, n = 4; LH, learned helpless animal, n = 4 rats, ***P* < 0.01, ****P* < 0.001, Student’s *t*-test.


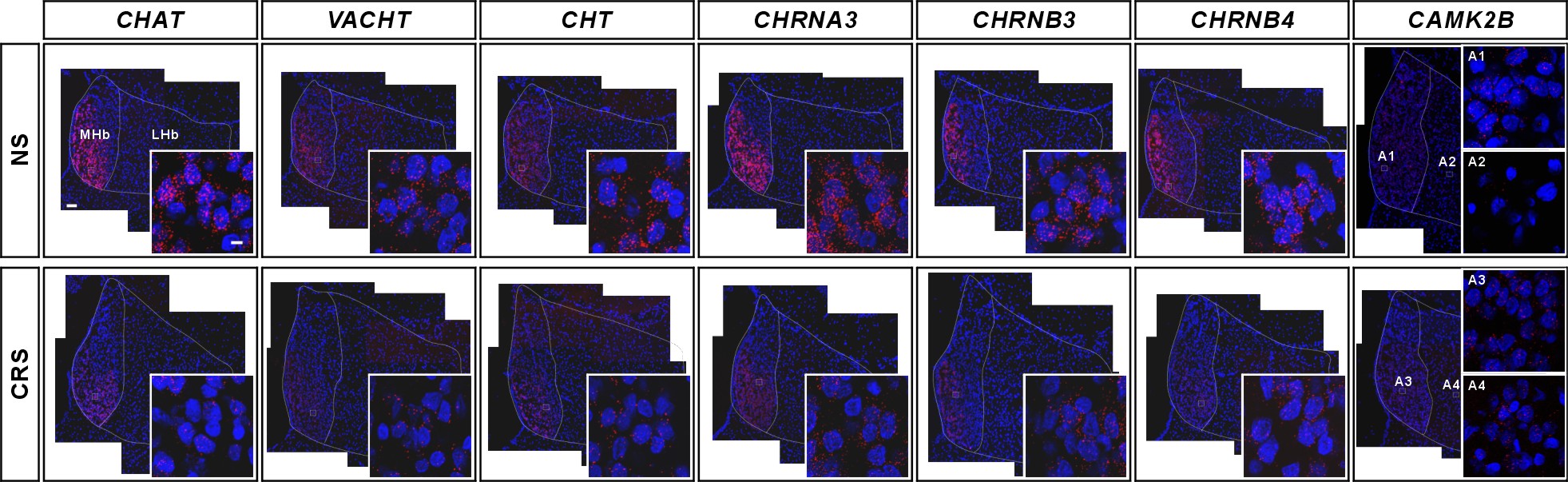


Figure S3. *In situ* RNA hybridization of CRS rats

RNAscope was performed on rat brain slices obtained from NS and CRS groups. Brain slices were hybridized with probes (red) for cholinergic genes (CHAT, VACHT, CHT, CHRNA3, CHRNB3, and CHRNB4) and CAMK2B. Slices were counterstained with Hoechst (blue). Inset boxes are higher magnification images of the original images. Scale bar, 50 μm; inset, 5 μm.


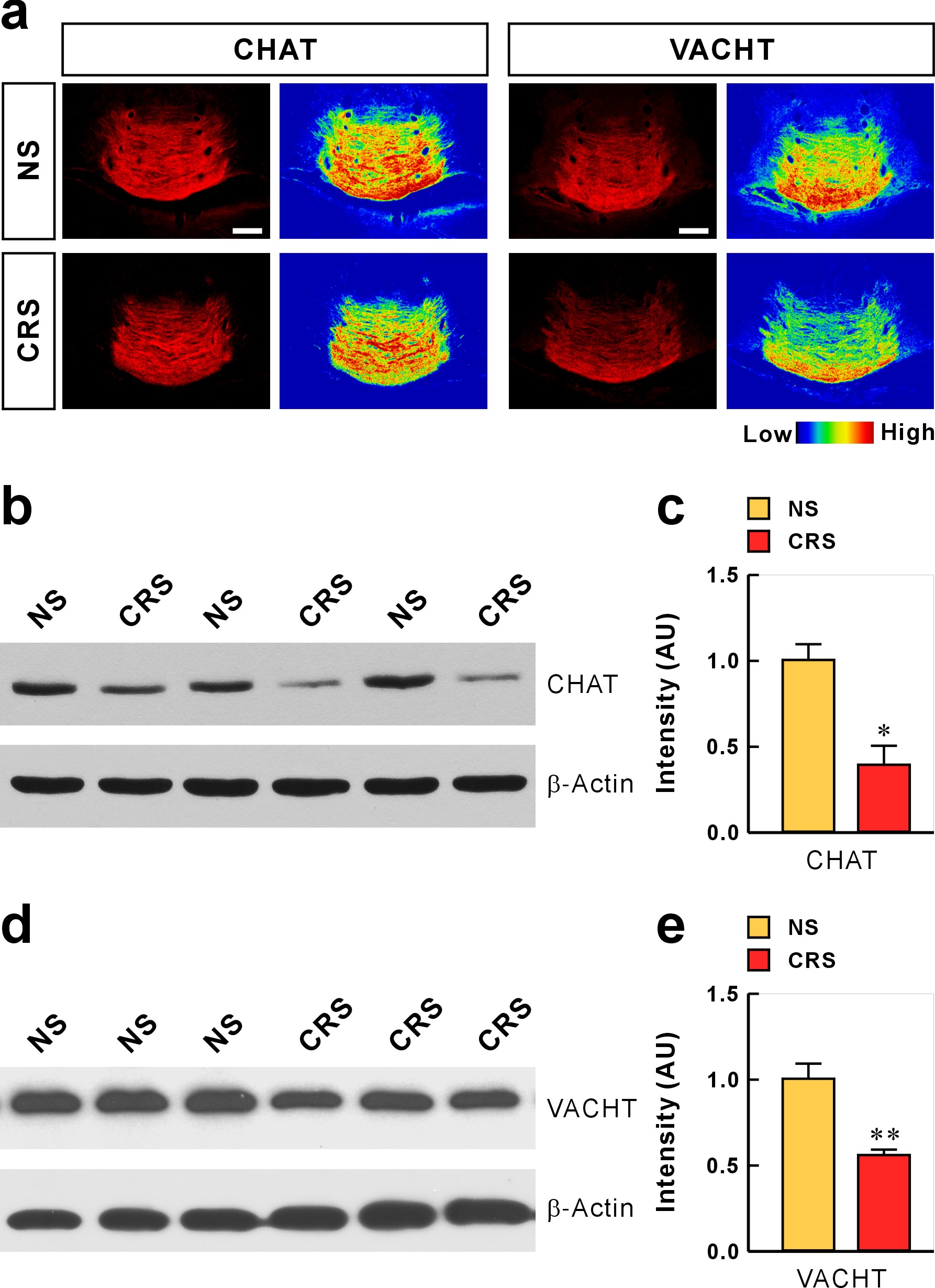


Figure S4. CHAT and VACHT expression in the interpeduncular nucleus of CRS rats

The protein expression levels were validated by immunohistochemical staining (**a**) and western blots (**b**−**d**) in the interpeducular nucleus exposed to CRS. Immunoreactivities of CHAT (**a**, left panel) and VACHT (**a**, right pannel) are shown in original (left columns) and pseudocolored images (right columns). In pseduocolored images, no detectable intensity level is indicated by *blue* and the highest level is indicated by *red*. (**b**) CHAT and VACHT western blots from IPN of rats exposed to CRS. (**c** and **d**) Quantification of the effects of CRS-mediated downregulation of CHAT and VACHT. Consistent with qPCR (Figure 1), the protein expression levels of CHAT and VACHT in the IPN were decreased in CRS groups compared to NS groups. Data represents mean ± SEM (NS, n = 3 rats; CRS, n = 3; **P* < 0.05, ***P* < 0.01, Student’s *t*-test). Scale bar, 200 μm.


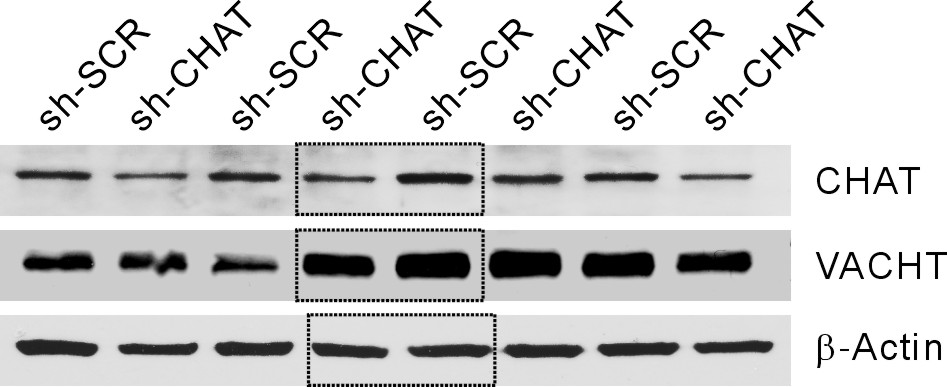


**Figure S5. CHAT knockdown reduced the protein expression of CHAT**

Specific knocking down of CHAT but not VACHT by the AAV-sh-CHAT. Boxes with dotted line are representative cropped images in Fig. 2e.


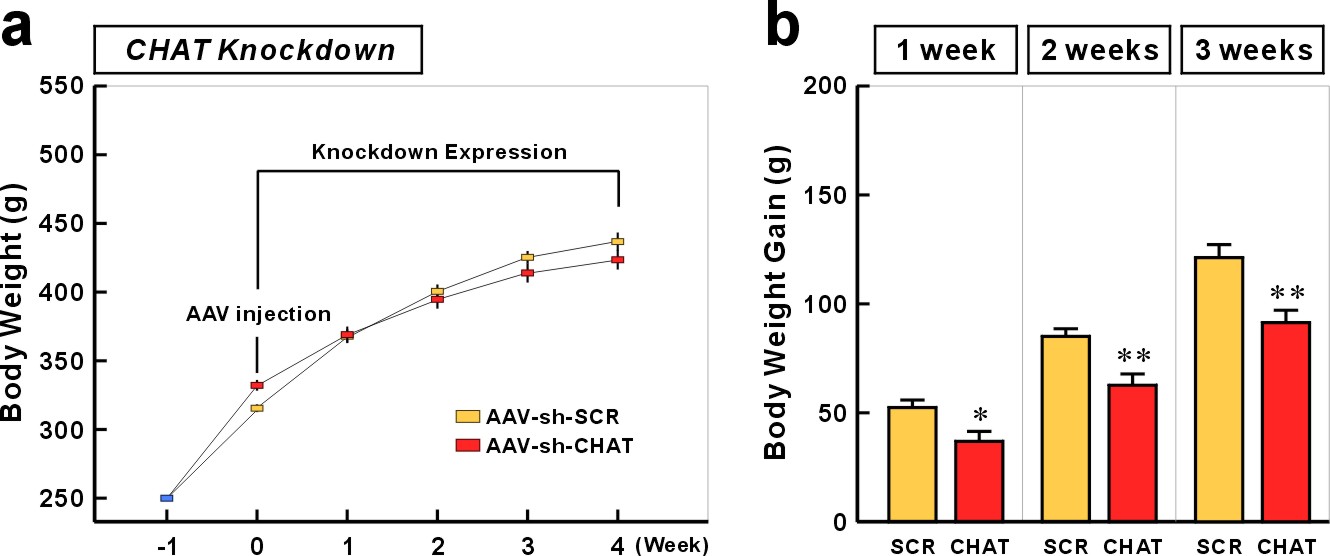


**Figure S6. CHAT knockdown in the habenula reduced body weight gain**

Infusion of AAV-sh-CHAT into the habenula induced weight gain defect. The rats were separated into two groups and AAV-virus was injected after 1 week habituation. (**a**) Body weight was monitored weekly for five weeks. (**b**) Weight gains were measured after AAV viral infusion. Data represents mean ± SEM (SCR, AAV-sh-SCR, n = 10 rats; CHAT, AAV-sh-CHAT, n = 20; **P* < 0.05, ***P* < 0.01, Student’s *t*-test).


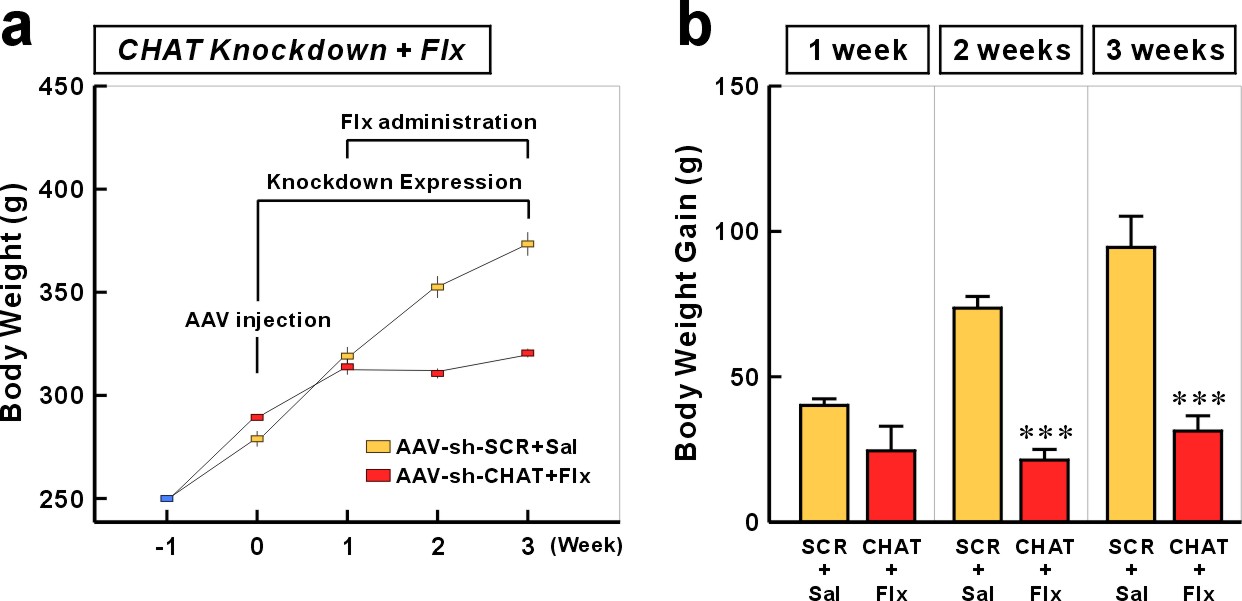


**Figure S7. Fluoxetine administration in the CHAT knock-downed rats reduced body weight gain**

Chronic administration of fluoxetine (Flx) of CHAT knock-downed rats over 2 weeks promotes severe weight gain defect. (**a**) Body weight was monitored weekly for four weeks. (**b**) Weight gains were measured after AAV viral infusion. Data represents mean ± SEM (SCR + Sal, AAV-sh-SCR + saline, n = 10 rats; CHAT + Flx, AAV-sh-CHAT + fluoxetine, n = 15; ****P* < 0.001, Student’s *t*-test).

**Supplementary Tables**

**Table S1. Primer sequences used for qRT-PCR in rat samples**

| ***Gene symbol*** | ***Primer sequence 5' → 3'*** | | ***Size (bps)*** |
| --- | --- | --- | --- |
|  | ***Forward primer*** | ***Reverse primer*** |  |
| SLC18A3 (VACHT) | AAACATCGTCCACTGGTCC | CTTTCCCTAAGATGCCTCCAC | 213 |
| SLC5A7 (CHT) | TTTCCAGATCCCAAGACCAAG | ATGCTCCAAACACAAACACAG | 253 |
| CHAT | TCCAAGACACCAATGACCAG | GGACGCCATTTTGACTATCTTTC | 350 |
| CHRNA3 | TTCTACCTGCCCTCCGACTGT | AATCCCGCCGTTCCTAAAATG | 594 |
| CHRNB3 | GAAAGGAGAGTGATACAGCCG | AGAAAGAGCCACAGGAAGATG | 223 |
| CHRNB4 | CTATCACTGTCCCAGCTCATC | AGAACCATGTCAATCTCCGTG | 377 |
| CAMK2B | CAGAAGCTGAGGGCCTCCCA | GGTCACAGATTTTCGCATAGG | 167 |
| GAPDH | CCTTCATTGACCTCAACTACAT | CAAAGTTGTCATGGATGACC | 497 |

**Table S2**. Efficiency data for housekeeping reference genes

| ***Gene symbol*** | ***Slope*** | ***R^2^*** | ***Efficiency (%)*** |
| --- | --- | --- | --- |
| GAPDH | -3.309475 | 0.9978 | 100 |
| TBP | -3.3670 | 0.99617 | 99.3 |
| CYC1 | -3.33036 | 0.99617 | 98.6 |

C_t_ values obtained from 3-fold serial dilutions of pooled cDNA were pooled against dilution factors. The reaction efficiency was calculated using the equation E = 10^(-I/slope)^, where E is the efficiency and slope is the gradient of the line of best fit. GAPDH, glyceraldehyde 3-phosphate dehydrogenase; TBP, TATA-box binding protein; CYC1, cytochrome c1.

**Table S3**. TaqMan probe-based gene expression analysis for human samples

| ***Gene symbol*** | ***TaqMan assay ID*** | ***Primers*** | ***Context sequence*** |
| --- | --- | --- | --- |
| SLC18A3  (VACHT) | Hs00268179_s1 |  | GTCATCGTGCCCATAGTGCCCGACT |
| SLC5A7  (CHT) | Custom | F: GAACATCTACCAGCTTTCCTTCAGA |  |
|  |  | R: TGATTCGCATAACCCAAACGATTTC |  |
| CHAT | Hs00252848_m1 |  | AGTGAGGAGTCTGGGCTGCCCAAAC |
| CHRNA3 | Hs01088199_m1 |  | ACCTGTGGCTCAAGCAAATCTGGAA |
| CHRNB3 | Hs00181269_m1 |  | TTGAAAATGCTGACGGCCGCTTCGA |
| CHRNB4 | Hs00609520_m1 |  | CCTTTGCGGGCGCGGGAACTGCCGC |
| CAMK2B | Hs00365799_m1 |  | AGCATTCCAACATCGTGCGTCTCCA |
| GAPDH | Hs99999905_m1 |  | GGGCGCCTGGTCACCAGGGCTGCTT |
| TBP | Hs99999910_m1 |  | GCAGCTGCAAAATATTGTATCCACA |
| CYC1 | Hs00357717_m1 |  | TCTTAGAGTTTGACGATGGCACCCC |

The context sequence is the nucleotide sequence surrounding the region to which the probe binds. The primer and probe sequences for the TaqMan assays are not available. Detailed information for each TaqMan assay is freely available at [http://www.appliedbiosystems.com](http://www.appliedbiosystems.com/). F, forward primer; R, reverse primer.
